# Fish Consumption and Blood Mercury Levels: Golding et al. Respond

**DOI:** 10.1289/ehp.1307997R

**Published:** 2014-05-01

**Authors:** Jean Golding, Colin D. Steer, Tony Lowery, Robert Jones, Joseph R. Hibbeln

**Affiliations:** 1 Centre for Child and Adolescent Health, University of Bristol, Bristol, United Kingdom; 2 National Seafood Inspection Laboratory, National Marine Fisheries Service, National Oceanic and Atmospheric Administration, Pascagoula, Mississippi, USA; 3 Inorganic and Radiation Analytical Toxicology Branch, Centers for Disease Control and Prevention, Atlanta, Georgia, USA; 4 National Institute on Alcohol Abuse and Alcoholism, National Institutes of Health, Department of Health and Human Services, Bethesda, Maryland, USA.

Obviously our article (Golding et al. 2013) must have been less than clear in leading Groth to assume findings that we had not claimed. For example, he states both that “there was no strong correlation between fish consumption and blood mercury levels” and that we “observed no association between fish intake and blood mercury.” Neither statement is true. We did show that the *R*^2^ for total blood mercury associated with seafood consumption was 8.75%, implying a correlation coefficient of about 0.3. The relationship between fish intake and blood mercury was highly significant (*p* < 0.0001).

The point that we were making in the article was that seafood did contribute to the total blood mercury levels, but that many other dietary items did so as well. The other studies quoted by Groth did not investigate other sources of mercury. However, two studies in the United Kingdom have shown that seafood provides only 25–33% of dietary mercury (Ysart et al. 1999, 2000); although we did not distinguish between types of fish, these authors assayed the mercury content of 500 different samples of seafood, typical of a normal UK diet.

In conclusion we do not disagree with Groth that excessive consumption of fish with high mercury content should be avoided, but would emphasize the overall beneficial effects of fish in general.
